# Expression of concern: Engineering the optical properties of nickel sulphide thin films by zinc integration for photovoltaic applications

**DOI:** 10.1039/d4ra90106a

**Published:** 2024-09-25

**Authors:** Junaid Younus, Warda Shahzad, Bushra Ismail, Tanzeela Fazal, Mazloom Shah, Shahid Iqbal, Ahmed Hussain Jawhari, Nasser S. Awwad, Hala A. Ibrahium

**Affiliations:** a Department of Chemistry, COMSATS University Islamabad (CUI), Abbottabad Campus 22060 Pakistan bushraismail@cuiatd.edu.pk; b Department of Chemistry, Abbottabad University of Science and Technology (AUST) Abbottabad Pakistan tanzeelafazal@yahoo.com; c Department of Chemistry, Faculty of Science, Grand Asian University Sialkot Pakistan; d Department of Chemistry, School of Natural Sciences (SNS), National University of Science and Technology (NUST) H-12 Islamabad 46000 Pakistan shahidgcs10@yahoo.com; e Department of Chemistry, Faculty of Science, Jazan University P. O. Box 2097 Jazan 45142 Saudi Arabia; f Chemistry Department, Faculty of Science, King Khalid University P. O. Box 9004 Abha 61413 Saudi Arabia; g Biology Department, Faculty of Science, King Khalid University P. O. Box 9004 Abha 61413 Saudi Arabia

## Abstract

Expression of concern for ‘Engineering the optical properties of nickel sulphide thin films by zinc integration for photovoltaic applications’ by Junaid Younus *et al.*, *RSC Adv.*, 2023, **13**, 27415–27422, https://doi.org/10.1039/D3RA04011A.

*RSC Advances* is publishing this expression of concern in order to alert readers that concerns have been raised over the integrity of the data published in this article. The authors have been contacted but have not provided the requested raw data. An expression of concern will continue to be associated with the article until a conclusive outcome is reached.

Laura Fisher

17th September 2024

Executive Editor, *RSC Advances*

